# A critical reflective analysis of patient and public involvement in a programme of heart failure with preserved ejection fraction research

**DOI:** 10.1186/s40900-026-00867-8

**Published:** 2026-04-17

**Authors:** Faye Forsyth, Peter Hartley, Jonathan Mant, Scott Rowbotham, John Sharpley, Alison Wood, Christi Deaton

**Affiliations:** 1 PACE Section (Perioperative, Acute, Critical Care and Emergency Medicine), Department of Medicine, University of Cambridge, Cambridge, UK; 2 KU Leuven Department of Public Health and Primary Care KU Leuven, Leuven, Belgium; 3 Clinical Academic Physiotherapist, Primary Care Unit, Department of Public Health & Primary Care, University of Cambridge, Cambridge, CB2 0SR UK; 4 Physiotherapy Department, Cambridge University Hospital NHS Foundation Trust, Cambridge, CB2 0QQ UK; 5 Department of Physiotherapy, The Queen Elizabeth Hospital King’s Lynn NHS Foundation Trust, King’s Lynn, UK; 6 Patient with Lived Experience of Heart Failure with Preserved Ejection Fraction Cambridge, Cambridge, UK; 7 The Healthcare Improvement Studies Institute, University of Cambridge, Strangeways Research Laboratory, Worts Causeway, Cambridge, CB1 8RN UK; 8 Primary Care Research, Primary Care Unit, Department of Public Health & Primary Care, University of Cambridge, Cambridge, CB2 0SR UK; 9 Primary Care Unit, Department of Public Health & Primary Care, University of Cambridge, Cambridge, CB2 0SR UK

## Abstract

**Introduction:**

There are very few reports of patient and public involvement (PPI) initiatives in cardiovascular disease research and even fewer in heart failure, the eventual end point of many cardiovascular conditions. This report describes and critically appraises a PPI endeavor conducted during a programme of work focused on designing a multi-component diet and exercise intervention for people with Heart Failure with preserved Ejection Fraction (HFpEF).

**Methods:**

This is a retrospective analysis and critical reflection of field notes and formal communication documents (produced during PPI meetings, *n* = 3) and transcripts (produced during workshops, *n* = 3) collected throughout the HFpEF research programme (PRESERVE-HFpEF).

**Results:**

PPI had a substantial effect on this project both in terms of the content of the intervention and on the researchers’ perceptions and skills in conducting meaningful PPI. For example, the dietary interventions envisioned within the intervention (caloric restriction, low carbohydrate and high protein), which were based on a meta-analysis of effects we (the authors) performed, were not deemed to be appealing nor feasible by PPI contributors. Many of the researcher related learning points we identified during the analysis, for example practical arrangements that maximize participation, allowing for more open dialogue and being mindful of scientific biases, were not obvious until they were subjected to an interrogative ‘critical’ process.

**Conclusions:**

To build a foundation of good practice and evidence of the potential impact of PPI in cardiovascular disease research, we need further descriptions of the involvement process. This reflection is one of very few reports describing how PPI was enacted in heart failure research and what the impacts were. We hope that it will provide other researchers with some insight into how (or how not to) approach and deliver PPI in their research journey.

**Clinical trial number:**

Not applicable.

**Patient or public contribution:**

People with heart failure with preserved ejection fraction were involved throughout the lifespan of this project. They participated in the PPI meetings and workshops where the direction of the research was discussed and their opinions and expertise were sought, initially through consultation type methods and later via consultation with co-production overtones. One member (JS) contributed to data analysis and interpretation; and provided critical feedback on emerging ideas and drafts of this report.

## Abstract (fr)

In this paper the researchers think back on the patient involvement activities they have conducted as part of a study that tried to create a new diet and exercise intervention for people with Heart Failure with preserved Ejection Fraction (HFpEF). HFpEF is the most common type of heart failure. It can be very difficult to diagnose and manage as the symptoms impact on quality of life and there are few treatment options. There is some evidence that suggests combined diet and exercise interventions can be beneficial to people with HFpEF, although the optimal combination remains unclear.

People with HFpEF who had been taking part in an ongoing study, were asked to contribute to the development and design of a combined diet and exercise intervention called PRESERVE-HFpEF. The researchers explain what they did to engage with people with HFpEF to shape the research idea and design of the intervention, and consider how their approach could be improved. They also explore what other researchers have said about involvement, and use this to show the weakness of their methods, or where they could have approached things differently.

This story of patient involvement shows how important good and meaningful involvement is, and the researchers explain how they missed opportunities to identify a very important belief that patients with HFpEF held. This belief was that diet intervention styles identified by a study of studies (meta-analysis), did not appeal to them and they would be unlikely to either take part in, or stick with, any future intervention that included these designs. This viewpoint, once realized, led to a complete change to the intervention. The contributor informed design focused on recommendations for small changes in shopping and eating habits that could be more easily achieved within everyday life, rather than having to follow complicated and detailed diet prescriptions.

## Background

Patient and public involvement (PPI) is increasingly a requirement of research supported by policy-makers and research funding bodies [1, 2]. However, there is marked variation across countries as to how PPI is prioritized and practiced [3, 4]. There also appears to be variable implementation of PPI across disciplines; with a recent review suggesting that cardiovascular disease research lags behind other fields [5]. Notable tensions within the cardiovascular disease arena include doubts about the value and impact of PPI [2, 6], uncertainty over the patient role, the scope of activities they should undertake, how best to support PPI contributors, and concerns about the representativeness of the patient voice [7, 8]. Challenges associated with delivering ‘meaningful’ PPI activities within restricted budgets and timelines, access to training, and a lack of agreed standards, have also been documented [9]. Cumulatively, these barriers can result in tokenistic or tick box approaches, which is quite often the reality of PPI in healthcare research [10, 11].

Heart failure is a condition that contributes significantly to the global burden of cardiovascular disease [12]. Heart failure with preserved Ejection Fraction (HFpEF), is now the most common sub-type of heart failure and is rising in prevalence, mirroring population increases in aging and multimorbidity [13]. HFpEF is described as very debilitating by people with lived experience, and it can be difficult to manage because of clinical complexity, heterogeneity and some uncertainty amongst the clinical community over its veracity and optimal management environment (cardiology versus gerontology) [14–16]. Complexity and uncertainty can be difficult to address, and patient voices are central to ensuring that any decisions relating to new care pathways or interventions, results in outputs that are relevant, accessible and attuned to their needs. As such, there have been increasing calls from patients, clinicians, policy makers and industry, to embed and prioritize heart failure patient involvement in research [17, 18].

Despite widespread acknowledgement of the value of involving people with lived experience, there are few, if any reports, of PPI in heart failure. There are a growing number of publications that report experiences and outcomes of co-designing care in cardiovascular disease [19], including in heart failure [20, 21]. However, co-design often follows distinct pathways and processes that differentiate it from consultation based public involvement in terms of the depth, extent and power dynamics [22]. Whilst lack of reporting does not necessarily translate to lack of engagement in PPI activities, it is nevertheless problematic as we do not know how PPI is understood, practiced, responded to and valued within the field of heart failure research.

Following a narrative review of the benefits of PPI, Brett et al. [23] called for more detailed descriptions of PPI initiatives in order to improve the evidence base of efficacy, and to drive improvements in practice [23]. Building on this work, Staley [24] outlined the need for more explicit exploration of the context (conditions or environment), mechanisms (how the PPI was done/methods) and outcomes (impacts) of PPI. In this paper we (the authors) report and critically reflect upon our experiences of PPI work, undertaken as part of a doctoral programme of research conducted in HFpEF (PRESERVE-HFpEF [25]). We considered this a necessary process to build the evidence base of the value of PPI in HFpEF research, and as a means to critically appraise our approaches in relation to current best practice and philosophical discussions surrounding the value and meaning of PPI [26]. We have used the GRIPP2 (Guidance for Reporting Involvement of Patients and the Public) reporting checklist [27] to structure this report, the completed GRIPP2 checklist can be found in the supplementary material.

## Definition of PPI

Our approach to PPI, although supported by a modest amount of research funding, was informal. Many of the now published standards, supporting documents [28], and frameworks [29], were either not available when we commenced our project, or we were not aware of them. Therefore, we did not pre-decide on a definition of PPI. Retrospectively, our position was most closely aligned to the National Institute for Health and Care Research (NIHR) definition of PPI: “research that is identified and performed ‘with’ or ‘by’ people with a condition, rather than ‘to’, ‘about’ or ‘for’ them” [30].

## Theoretical underpinnings

Similarly, we did not specify the theoretical underpinnings of our approach. Reflecting back, our theoretical position would be defined as consequentialist. Based on Greenhalgh’s definition [29], consequentialist argue that PPI brings a ‘real-world’ and ‘lived-experience’ view that “improves the efficiency and value of research via a number of mechanisms.” These mechanisms include, but are not limited to, increased relevance to patients, improved recruitment, retention, dissemination and wider impact.

## Aim

To summarize, using the GRIPP2 reporting recommendations [27], a PPI initiative conducted during a programme of doctoral work that focused (PRESERVE-HFpEF) [25]. A secondary aim was to use a critical reflective approach [31], to reflect upon and learn from the experiences.

## Methods

### Design

**Fig. 1:**
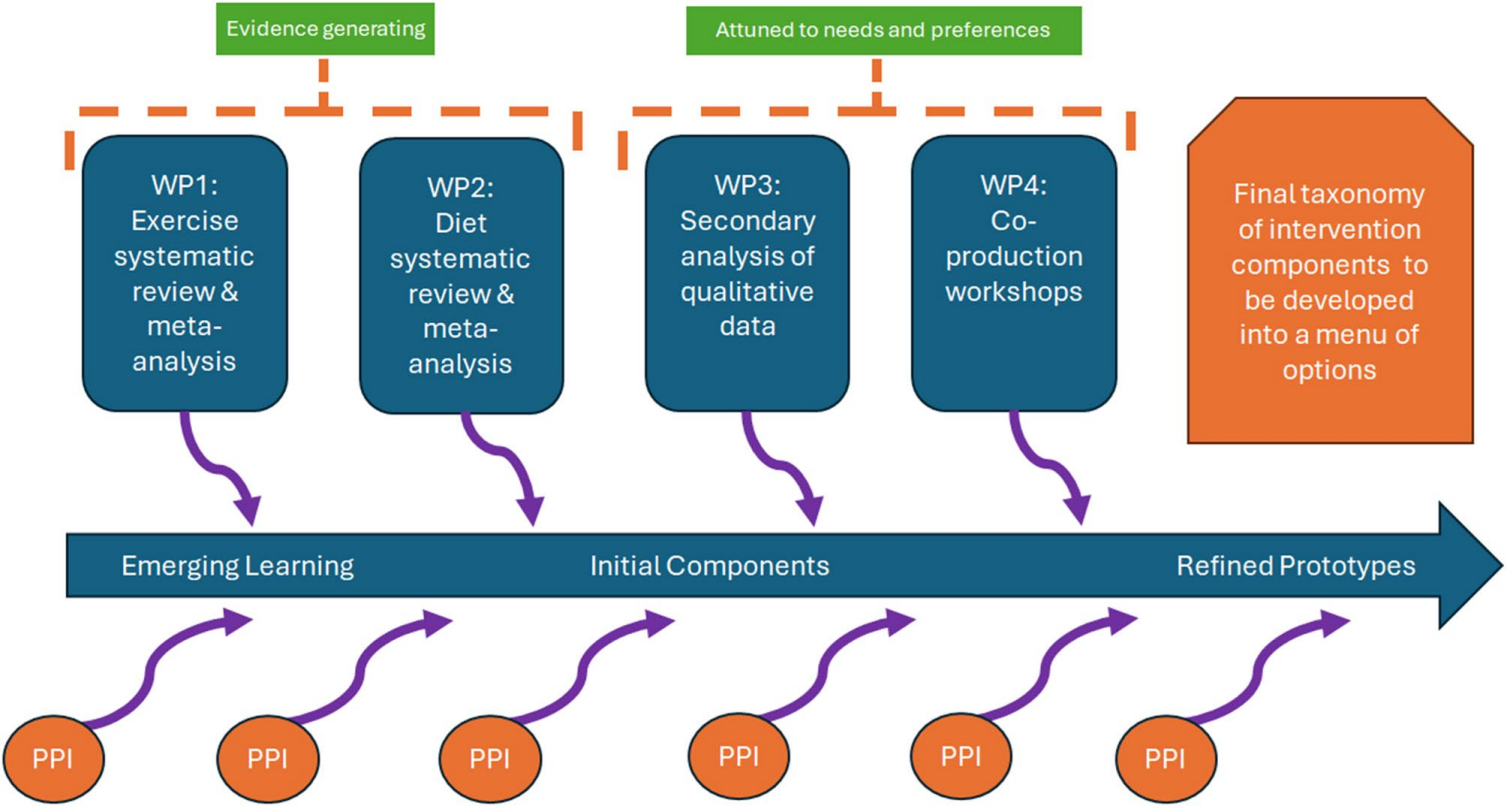
Schematic of doctoral programme of work with embedded PPI

This is a retrospective analysis with embedded critical reflection. Documents relating to PPI activities that were embedded in the PRESERVE-HFpEF doctoral programme of work were imported into NVivo 12 for management, then ordered and annotated to produce a descriptive narrative of events. A visual summary of the doctoral programme situating the PPI is provided in Fig. 1. The work packages have been reported separately (WP1 exercise reviews [32, 33], WP2 diet reviews [34, 35], WP3 secondary qualitative analyses of the lived experience [14, 15], WP4 co-production work [36]). This description focusses solely on the PPI activities undertaken and employs critical reflection methods to distill learning. Meetings were held according to project needs between 2018 and 2024, the reflection was undertaken between workshops 1 and 2 and finalised after workshops 2 and 3.

**Fig. 2:**
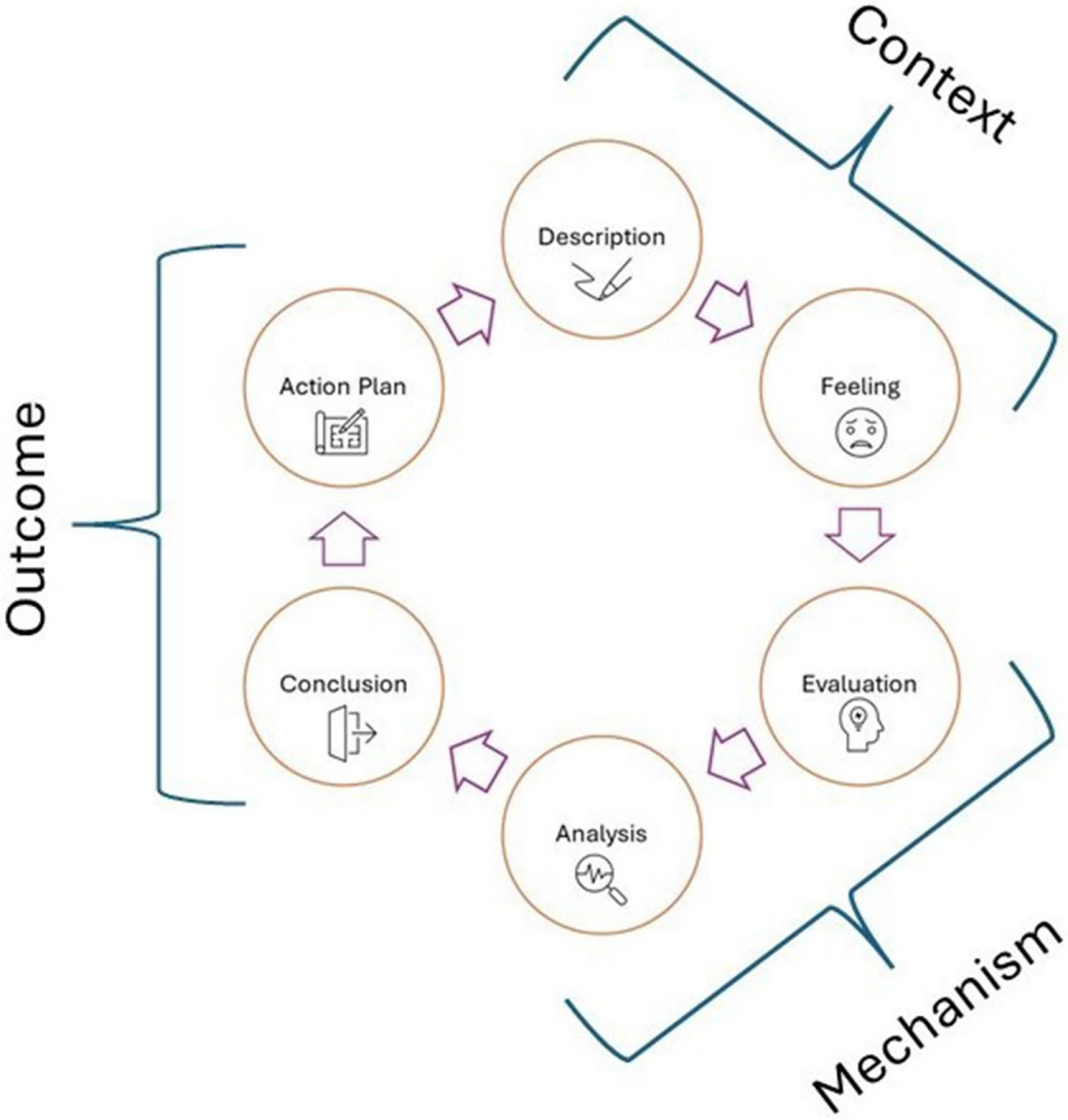
Gibbs’ reflective cycle and context, mechanism, outcome principles

Gibbs’ reflection cycle was used to guide the reflection process [31]. This model focusses on describing what happened, thoughts and feelings surrounding the event, evaluating what was good/bad about the experience, analyzing the event by drawing on external sources (i.e. the literature), drawing conclusions and creating an action plan or summarizing the learning [31]. For each PPI event within our narrative, we progressed through Gibbs’ reflective cycle and documented our descriptions of the six stages. We linked each stage of the reflective cycle to the context, mechanism, and outcome principles described by Staley [24]. We defined stage 1 and 2 of our reflection process as identifying the context of our PPI, stage 3 and 4 as interrogating the mechanisms and stage 5 and 6 as exploring of positive/negative outcome (Fig. 2). We regularly revisited the original data to confirm, refute or reconsider the properties of our descriptions, particularly during the analysis stage when we consulted the wider literature to make sense of, test or compare our descriptions to that recorded in the wider PPI evidence base.

The reflection principally mirrors the first authors’ perspective, however all authors who were in attendance at the PPI activities provided feedback based on their recollections and positionality. The first author acknowledges her standpoint as an educated, white British, female researcher, with a research interest in HFpEF and a belief that researchers are not attuned to needs and wishes of people living with the condition. The first author acknowledges that this positionality may have influenced the reflection and subsequent presentation.

### People involved

Multiple sources of data relating to PPI work conducted over the six-year period were collated. These included contemporary field notes from PPI group meetings, formal communication documents (i.e. letters, emails, PowerPoint presentations) created before and after these meetings, and three transcripts from workshops. Early meetings involved both people with HFpEF and those with chronic conditions. The participants with chronic conditions came from Cambridge University Hospitals Patient and Public Involvement panel and were co-opted to initial meetings as it was felt their experience would support novice PPI contributors. The workshops included PPI group members, supplemented by additional people with lived experience of HFpEF recruited from an earlier programme of work [37], ethical approval was obtained to hold the workshops as they were recorded and transcribed verbatim, informed consent was also obtained. One patient (JS) with specialist knowledge of HFpEF expressed a broader interest in involvement and consented to be consulted ad hoc, and to participate in analysis/report writing. All meetings and workshops were supported by researchers with clinical expertise in heart failure and exercise, but not PPI. See Table 1 for a detailed description of people involved.

**Table 1:** Overview of attendees at meetings and workshops

Meeting or workshop	Attendees
Meeting 1, November 2018	HFpEF *n* = 3, chronic conditions *n* = 2, researchers *n* = 2.
Meeting 2, April 2019	HFpEF *n* = 4, chronic conditions *n* = 2, researchers *n* = 4.
Meeting 3, April 2020	HFpEF *n* = 8, researchers *n* = 4.
Workshop 1, August 2022	HFpEF *n* = 2, researchers *n* = 1
Workshop 2 and 3, June 2023	HFpEF *n* = 7, spouses *n* = 2, researchers *n* = 5

### Stages of involvement

The PPI activities spanned the period from study inception in 2017/18 (development of the grant proposal to submit to funding bodies), through analyzing and interpreting interim findings in 2022/23, to study conclusion in 2024 (taxonomy of diet and exercise intervention components for HFpEF.)

### Level or nature of involvement

Four broad categories of involvement in healthcare have been described [38]: nominal (where involved partners confirm the legitimacy of a project); instrumental (which focuses on efficiency factors like recruitment); representative (that aims give the target audience a voice; postulated to enhance sustainability); and transformative (whereby empowerment is a key goal). These would equate to NIHR descriptions of consultation (nominal and instrumental), collaboration (representative), co-production and user-controlled research (transformative) [30]. In practice most researchers will draw flexibly across these types of involvement [30, 38]; as our project evolved, we moved from a nominal approach through a representative approach to an eventual transformative level.

Although not directly utilized, our PPI activities broadly adhered to the UK Standards for Public Involvement [39]. A broad range of people with lived experience of HFpEF, enrolled in an established programme seeking to optimize HFpEF management in primary care [14, 37, 40], were offered the opportunity to join the PPI group. All participants who agreed to take part provided consent that included agreement to the storage and processing of their personal identifiable data for the purposes of continued contact and expense reimbursement. To address known barriers such as transportation, we provided transport or reimbursed expenses. To counter obstacles unique to this group of older adults with HFpEF and multimorbidity (breathlessness, frailty); we provided mobilization support. Meetings were held in comfortable conference rooms and refreshments were provided. Time at the beginning and end of the meetings were dedicated to socializing, which was voiced as important during meetings. No payment for time was offered, however attendance was not mandated, participants could engage flexibly, and they had the opportunity to keep any of the exercise equipment they trialed during sessions (subject to physiotherapist suitability and safety assessment).

As the group evolved, we employed different tools and techniques, meetings with/without an agenda, use/non-use of PowerPoint presentation, directed work (i.e. annotating study documents) or free discussion. We also invited guest speakers to address informational needs identified by the group; these were generally non-PPI related, rather support needs associated with their disease. Opportunities for more advanced involvement were offered, this was pursued by only one participant [14, 41]. We communicated regularly via letter, including summarizing meeting content and sharing resources requested. Further, we conveyed how the views of the PPI group had been acknowledged, or how they had affected the subsequent work. The latter suggests guideline adherent, effective PPI. However, it is only through summarizing our learning via a critical reflective approach (see results) that a more evaluative report can be generated.

### Capture or measurement of PPI

There is no consensus regarding the optimal methods to capture and measure impact [42]. Some argue that the experiential knowledge generated, for example the emotional impact on the researcher, cannot be measured empirically [43]. Others contend robust quantitative measures of impact are needed to strengthen the evidence base [44]. We captured our PPI activities using a range of methods including field notes, letters and PowerPoint presentations for the PPI group, and transcripts from digital recordings. These sources, and accompanying reflections, constitute our measurement methods. In terms of robustness, field notes and letters would be considered comparatively weak, as these would have relied upon field notes and memory [45]. Slides presented during the meetings and transcripts of digital recordings are more rigorous sources of data. Critical reflections based on the data were post-hoc, therefore subject to recall bias.

### Economic assessment

We did not undertake any economic assessment therefore cannot report on this criterion.

## Results

### Face to face PPI group meeting 1 – consultation on results feedback preferences

***Description and feelings (context)****:* The aim of the meeting was to seek advice on feedback preference for a now concluded programme of work called OPTIMISE HFpEF [37]. The PPI group were asked for their views on the optimal format for sharing research information (paper versus digital). Mixed opinions were expressed, contingent upon access to, and competency with, information and communication technology. The group digressed to debate information provision in healthcare settings more generally; they considered how this is often not forthcoming. PPI group members with HFpEF described how their diagnosis had not been clearly communicated, nor had they received management advice. Consequently, they reported poor knowledge of the disease and self-care strategies. Heart failure self-care information was accessed online; information specific to HFpEF was difficult to retrieve and attendees asserted it did not meet their needs.

After the meeting, literature relating to two core components of self-care (diet and exercise) were explored further with a view to providing summaries of self-care strategies in the post meeting feedback letter. Database searches on these topics returned limited results. Thus, feedback provided during the consultation inspired a new programme of research (PRESERVE-HFpEF) that would 1) address the information gap expressed and 2) close the research gap identified in the literature review. At the time, we considered that the information garnered on communication methods justified the continued use of paper based communication in the core project (OPTIMISE HFpEF) [37]. We also considered the new project (PRESERVE-HFpEF) to be a legitimate research priority, as people with lived experience had spontaneously directed the research and endorsed this as a patient need.

***Evaluation and Analysis (Mechanism):***That we considered the new project to be a research priority based on one meeting that involved a small group of individuals, is problematic. It is important to remember that as researchers we bring a ‘curated picture’ of the clinical problem [46], one that is shaped by the academic publications we have read and our patient encounters (including PPI events). What we prioritize, no matter how genuine our we believe these priorities to be, may not always be what matters to people with lived experience and it is important to check-back before making significant decisions [6].

***Conclusions and Lesson (Outcome)****:* Researchers may make extrapolations and assumptions without realizing it. A reflective approach is key to improving the practice of PPI, as it can help us identify when we might be making an unfounded decision. In retrospect we feel we conflated need with priority; just because the participants agreed that something might be useful, does not mean it is a priority for research.

## Face to face PPI group meeting 2 – consultation on lay summaries and research equipment

***Description and feelings (Context):*** During this meeting, a plain English summary of the PRESERVE-HFpEF project was circulated, the research facilities were viewed, and some exercise capacity measurement equipment was trialed for suitability. Feedback from group members resulted in some changes in the project, notably the group thought cardiopulmonary exercise testing on a bicycle was preferable to a treadmill. People with lived experience were consulted, their feedback and preferences were obtained, and changes to the funding proposal were made in recognition of the preference for bicycle based cardiopulmonary exercise testing. At the time we regarded this a positive outcome.

***Evaluation and Analysis (Mechanism):*** With the benefit of our reflective lens, the approach could have been improved. Knowles et al. [6] have described the importance of ‘space to talk’, which refers to allowing room for dialogue, disagreement and patient expertise within any PPI activity. The group were shown equipment and asked which they would prefer. We did not allow sufficient space to discuss alternative options for measuring exercise capacity (i.e. six-minute walk test); nor did we allow the group to debate the pros and cons of various methods which might have allowed for more informed decision making. Allowing for space to talk about alternative methods for exercise testing, could have enabled a more robust assessment of the possible approaches, thus enhancing the acceptability and feasibility assessment from the contributors.

***Conclusions and Lesson (Outcome)****:* Greater two-way interaction should be the norm in PPI, not the exception. As Knowles et al. [47] assert, the researchers’ contribution should be as the ‘catalyst for discussion’ not the arbiter of it. Lack of time is regularly documented as a barrier to delivering PPI, for it not only prohibits space to talk but also relationship forming, trust building, team cohesion and in some cases, the necessary culture change required to facilitate meaningful PPI [10]. The main learning point was that presenting an already established programme of work (as we did), is problematic, as it is not offering space to talk about what should be done and how it could be delivered. It is possible that allowing for space to talk would have highlighted problems with the intervention design that only came to light much later our PPI journey (see workshop 1, 2 and 3).

## Face to face PPI group meeting 3 – consultation to obtain feedback on qualitative studies

***Description and feelings (Context):*** The third meeting focused on reviewing findings from now published qualitative studies [14, 40]. The group were led through a presentation describing three cases from the qualitative studies, each contained anonymized clinical details, direct quotations reflecting what had been said during interviews and summaries of what we, the researchers, believed the data demonstrated. During the meeting we asked the group questions about the validity of our interpretations i.e. did they think our interpretation was a fair and true reflection of what had been said.

***Evaluation and Analysis (Mechanism):*** We did not allow for in-depth participation in the data analysis, rather we asked the group to confirm the validity of the findings. Other researchers have successfully provided training and facilitated PPI participation in qualitative research analysis [48–50]. There is also evidence to suggest this can even be done rapidly [51], which is often the rationale to *not* pursue collaborative data analysis. Further, a framework for the this has been developed, based on the collective lessons from multiple studies [52]. As such, there is precedent and guidance available to facilitate this type of involvement.

***Conclusions and Lesson (Outcome)****:* Previous research has documented tensions relating to the involvement of people with lived experience in academic processes, like qualitative data analysis, that require ‘technical research skills’ [11]. However, the availability of guidance and plethora of previous success stories, suggests this should at least be considered. In hindsight, we have come to realize that endorsement is not really involvement. True involvement requires more time to enable reflection and fuller participation.

## Workshop 1 – translating research findings on diet into intervention components

***Description and feelings (Context):*** The following months were spent contemplating what our academic findings meant for the future intervention. The scoping review [35] and meta-analysis [53] suggested that diet may improve outcomes, however, the interventions tested were complex and likely incompatible with the capacity and capability challenges described by people living with HFpEF in the qualitative study [14]. We decided to explore this tension further in a small workshop with available members of the core PPI group. At this event we presented the findings from the reviews, in brief and in lay language, and asked them what they might mean in terms of developing the intervention. The contributors reflected upon their experiences of attempting and sustaining dietary change and reported how challenging this was in the context of multi-morbidity (conflicting advice), caring for others and costs. We considered this approach an evolution of what we had done before, for we were not seeking endorsement of an already defined approach, and we were allowing for open dialogue.

***Evaluation and Analysis (Mechanism):*** Guidance recommends the use of plain language in PPI activities to ensure the approach is accessible and appealing [30]. This approach introduces a potential parallel issue, that of oversimplification. Oversimplification of textual information is challenging because it is viewed negatively by the public [54]. Further, it risks continuing models of power whereby the people with lived experience are considered passive recipients [55] or consumers [56] of information. Failure to recognize or address imbalances in power relations is a commonly reported barrier to effective PPI [2, 10, 56]. We created very simplistic slides of our findings without considering the impact this may have had on the power dynamic within the workshop and subsequent discussion.

***Conclusions and Lesson (Outcome)****:* It can be difficult to strike the right tone when sharing technical research information with PPI group members, which should be inclusive without being infantilizing. As Locock et al. [2] state, researchers hold most of the cards and they shape and control how people get involved. Therefore, we need to reflect on our own position of power and make sure we are not making assumptions about capabilities which might moderate participation. With hindsight we appreciate that paternalism can be limiting and that not everything needs to be distilled to its most simplistic level. In doing so, it may be regulating the contribution of people with lived experience. A lack of appetite for dietary change was voiced in this workshop, however only at the end when discussing the project more generally. If we had presented the findings in more detail, we could have identified that the diet change components that we envisioned delivering in the future intervention, were not appealing nor feasible for people with lived experience.

## Workshops 2 and 3 - translating research findings on diet and exercise into intervention components

***Description and feelings (Context):*** In the first workshop participants described that, whilst they would be receptive to receiving dietary advice, they would be unlikely to implement dietary changes. Despite the clarity of this message, we were reluctant to discard a dietary component from any future intervention for two reasons. Firstly, we believed the scientific evidence implicating diet in the pathogenesis, prognosis and management of HFpEF, warranted its inclusion [35]. Secondly, we had some concerns about the representativeness of the previous event given the small sample (*n* = 2). A larger PPI workshop, that included core PPI group members and additional people with lived experience HFpEF, was therefore scheduled. During the event organization process, it became clear that there would be too many participants present to enable deeper discussion. Therefore, additional moderators were co-opted and the event was split into two (workshop 2 and 3). During this event we shared more extensive information on our previous findings (diet and exercise meta-analyses) and again asked what this might mean for the design of a future diet and exercise intervention.

As with the preceding workshop, the overall position of the group was that advice was welcome, but that dietary change was difficult. When pressed, they agreed that the complexities and unintended consequences of dietary change (altered bowel habits, medication interactions, conflict with other diet advice provided for concurrent conditions), meant that implementation was ultimately unlikely. In terms of exercise the participants recounted multiple barriers, suggested potential facilitators that would motivate them and important content that would be acceptable to them. A full report of the exercise preferences has been published [36].

The feedback indicated a potential tension between literature-based evidence, patient experiences and desired components and conditions for an intervention. For example, review evidence suggests whole scale dietary change approaches that reduce kilocalorie intake, carbohydrate intake or increase protein intake may improve outcomes. However, this was considered burdensome and incompatible with the capabilities of people with HFpEF and what they consider to be acceptable (accessible, information giving approach.) Overall, the conclusion from this meeting was that a substantial rethink was needed to harmonise evidence-based recommendations with desired intervention components by providing these in the suggested format (bite-sized information) and in a stepwise manner that allows for incremental increases over time depending on skill, safety and tolerance.

**Workshop Photos** (obtained and reproduced with permissions) 

Workshop members interacting during a comfort break
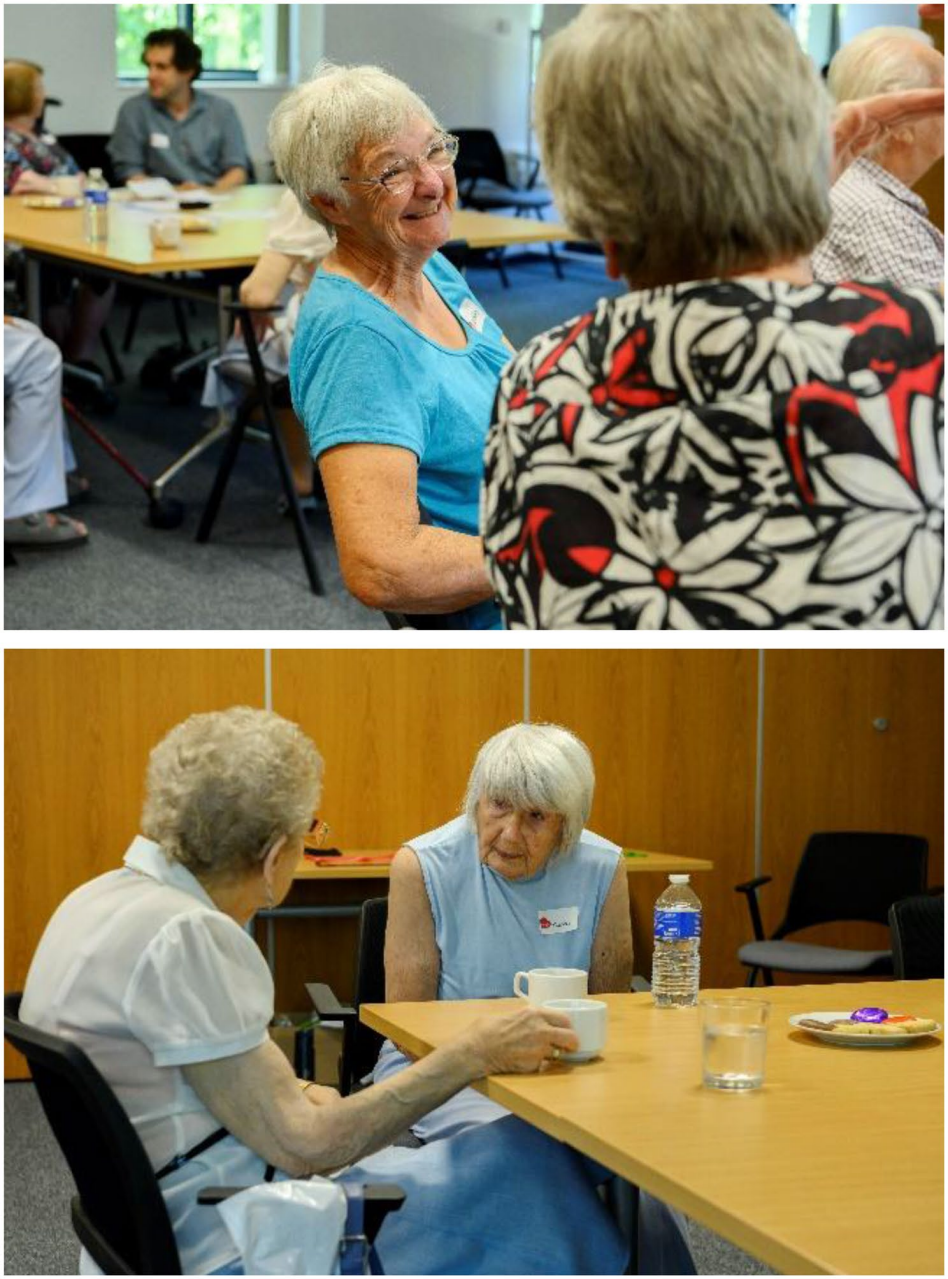


**Figure:**
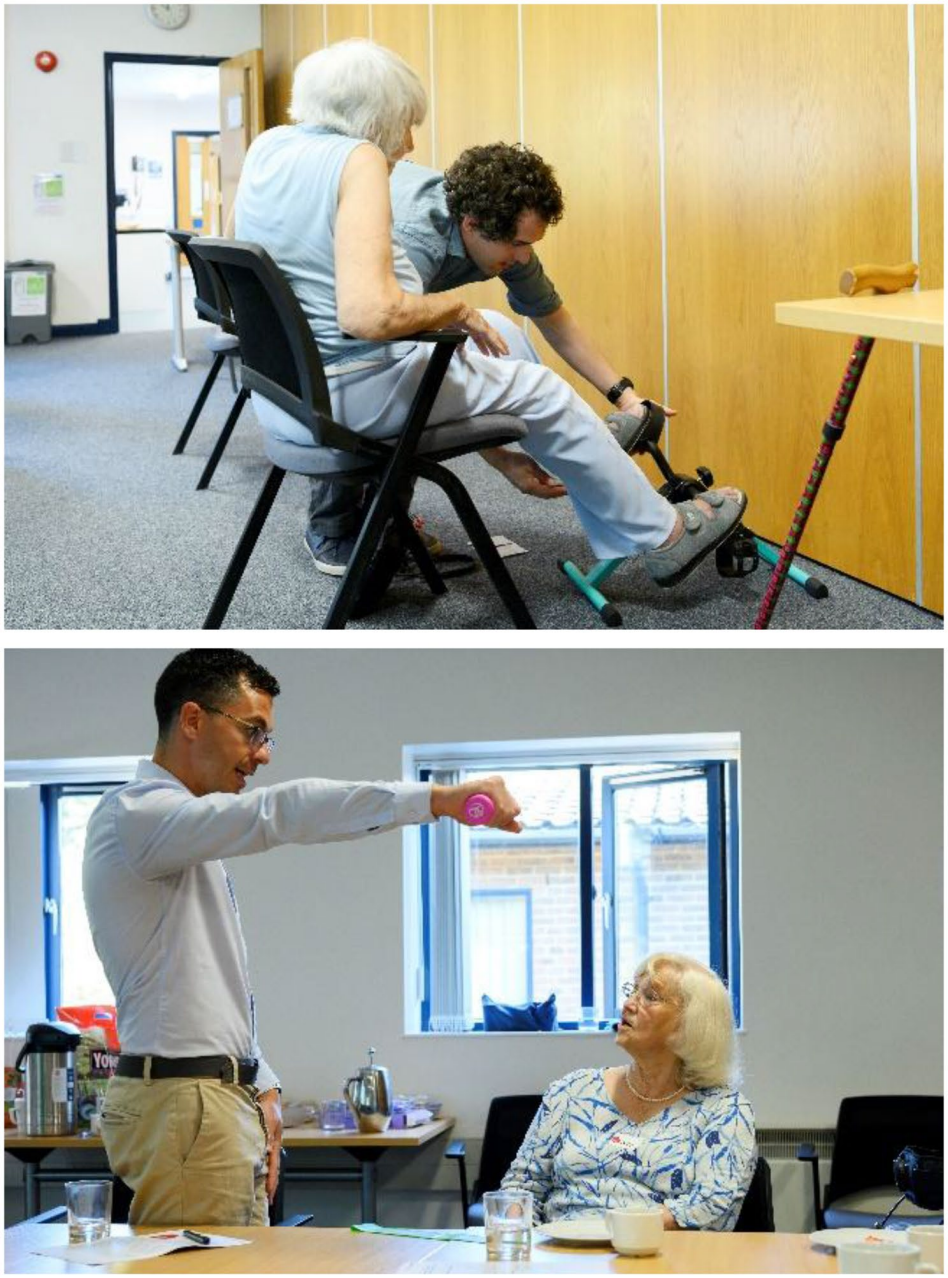
Workshop members receiving demonstrations of exercise equipment

*Evaluation and Analysis (Mechanism):* This meeting highlighted an important tension between the conduct of academic research with the incorporation of the views of patient. Our approach was biased towards science and academic criteria of robustness and validity. McGuire and Britten have extensively explored forms of patient representativeness [55]. They concluded that, whilst it is possible to describe different conceptualizations of representation within published research, in reality these are ‘nebulous and shifting’. Instead, they suggest asking ‘whether an act of representation is bogus’. Tensions between empirical and experiential knowledge have been documented before [23, 57]. However, only a few reports have described relatively substantial changes in their design [58, 59]; such as was recommended by our PPI group. Knowles et al. [47] have described the importance of ‘space to change’, which they define as space to adapt in response to contributor feedback.

*Conclusions and Lesson (Outcome):* Our bias toward the scientific research we had generated meant that we questioned the validity of the feedback we had received due to concerns about academic representativeness, and continued to push forward our research agenda on the basis that it was justified by the scientific evidence. In our case, the conflict between science (diet change is warranted) and experience (diet change is unlikely) was quite significant and has necessitated a rethink in how we should progress the development of the intervention. From this event we have learnt that researchers need to consider their biases and how they might influence PPI activities. When a position is contradicted, whether biased or not, we must be flexible and adaptable to patient feedback. In retrospect we could have decided that, based on workshop 1, the diet intervention we had conceived was not appealing and therefore spent more time in workshop 2 and 3 exploring what this meant and how the intervention should change as a result.

## General learning across PPI meetings and workshops

For researchers naïve to PPI in community dwelling older adults with HFpEF, the following practical recommendations, generated from across consultations and workshops might help ensure the comfort and wellbeing of those involved (Table 2).

**Table 2:** General recommendations to support the involvement of people with HFpEF

Practical Recommendations	Rationale
Transportation support	People with HFpEF may have limited transportation options, particularly in rural areas. Providing comfortable door-to-door transportation may enable greater participation. Arrangements should be clearly communicated and a support telephone number provided in case of any problems.
Accessible venue and meeting room	People living with HFpEF can have multiple comorbid conditions in addition to HFpEF and/or functional limitations that can make mobilizing challenging. Some people may require long term oxygen therapy, or wheelchair transfers - all reasonable accommodations should be made to support the participation of people with functional impairments. For example, we met contributors at the drop off location and supported them to mobilize.
Accessible, adjacent toilets	People living with HFpEF are often prescribed diuretic therapy which can be associated with urinary urgency and frequency, accessible and adjacent toilets can reduce anxiety, enhancing inclusiveness.
Co-morbidity compatible refreshments	People with HFpEF can have comorbid Type 2 diabetes or other conditions that necessitate adherence to a specific diet, these should be ascertained ahead of any activities and catered for.
Medication adherence support	Diuretic and other guideline directed treatments for HFpEF should be considered and accommodated within any activities. Delaying diuretics or other therapies during activities can have adverse health effects, particularly in the post-decompensation recovery phase.
Regular breaks and refreshments	People with HFpEF often experience fatigue, regular breaks should be scheduled to prevent over-fatigue that might have consequences in subsequent days (compensatory rest days).
Social time	People living with HFpEF can experience loneliness and social isolation, building in social time is beneficial for rapport building, it can also be motivating.

## Discussion

### Overview of findings

This work illustrates the value of PPI in healthcare research and the importance of improving researchers’ awareness, active engagement with, and reflections on, PPI activities. The impact of the PPI activities on this project is substantial, as the future intervention will approach dietary change entirely differently, as directed by the PPI participants. This is despite the evidence of health gains that result from some dietary interventions in HFpEF like caloric restriction and carbohydrate restriction, which we generated as part of this project [53]. The strength of feeling and level of consensus amongst PPI contributors was such that we feel we have no mandate to include any components that would require the level and degree of dietary change needed to affect the types of benefits identified in our meta-analysis [53]. Further, to do so would be to contribute to research waste as the challenges related to dietary change would ultimately mean inability to implement or sustain the intervention. Instead, a more nuanced approach is needed whereby information giving and practical assistance or advice is offered and perhaps built upon, incrementally reaching the large adjustments identified in the meta-analysis, if tolerated.

If at the outset we had focused more robustly on existing PPI literature, pre-emptively considered the theoretical underpinnings of our approaches, engaged with existing public involvement frameworks, and designed PPI activities based on this knowledge; we feel we could have identified this conflict between scientific and experiential knowledge earlier. Multiple public involvement frameworks have been developed, most of which are project specific [29]. In line with recommendations from Greenhalgh *et al.,* we would recommend researching previous framework and developing a bespoke PPI structure via their build-your-own framework template. Moreover, we could have reconsidered or adapted our approach sooner, i.e. capitalized on the knowledge rather than revisiting it. Contemporary research has since confirmed that there is discordance between clinicians and patients in the prioritization of dietary management within heart failure. In a descriptive study, Min et al. [60] compared learning needs ranked by patients and clinicians; they reported that clinicians rated dietary management advice as a higher priority than patients.

As with much PPI, one of the largest impacts has been on the researcher team [46, 47]. Not only are we now more aware of our own prejudices, but we have a deeper understanding of how we can do things differently going forward. Staley et al. [61] have extensively described how learning is an outcome of significant importance in involvement research, both for the PPI contributors and the researchers themselves.

### Comparison with heart failure PPI literature

There are few reports of PPI work in heart failure research and even fewer which explicitly document fundamental changes such as described here. Vroonland et al. [9] examined what impact researcher consultation with a committee of patient experts with cardiovascular disease had on subsequent grant submissions. They reported that whilst some changes were implemented (40% of PPI recommendations); these tended to focus on language and information within patient documents or the extent of patient involvement. Substantial changes to design or conduct did not tend to be incorporated [9]. This contrasts with our approach, where substantive changes have been made.

Within heart failure specifically [17], a literature review of transitional and self-care care programmes followed by a round table discussion involving patients, clinicians and industry partners, determined that patients were ‘not integrally involved’ in interventions designed and tested to date, and that there was significant scope for improvement. Our work contrasts here again, as we embedded regular PPI activities, learned from them and responded to recommendations by changing the project.

Lastly Newhouse et al. [62] reported on PPI work they undertook to assess whether changes were needed in the design, intervention, methods or outcomes of a heart failure toolkit intervention they had tested in urban centers and intended to transfer to a rural setting. As a result of their engagement work, changes were made to the protocol to enable inclusion of a specific patient reported outcome measure. Like this evaluation, we identified a need to capture different outcomes to reflect what is important to people with HFpEF in the context of a multi-component intervention (see companion publication [36]).

### Comparison with doctoral research PPI literature

Accounts of PPI in doctoral work are beginning to appear that validate findings here. For example, Dawson et al. [63] have described how their PPI work ‘filled gaps in the researchers experiential knowledge’. Troya et al. [64] contend that PPI activities in a mental health doctoral programme of work resulted in improved relevancy, legitimacy and validity of findings.

### Critical perspective and limitations

The key reflections as outlined in the results, were not obvious until they were subjected to an interrogative ‘critical’ process. Other researchers have spoken of the importance of reflection within PPI and published critical reflections have generated important insights which others can learn [46, 64, 65].

However, this research is not without its limitations. The reflections were post-hoc, substantially so for early meetings, introducing recall bias. The report is based predominantly on one researcher’s perspective, whose positionality could have influenced the description and learning. Lastly, variable quality documentary evidence was utilized, particularly so for field notes where there is an interpretation of an interpretation. All PPI contributors were of white British ethnicity and were recruited from a single locality in the east of England (Cambridgeshire), therefore, whilst we believe their voices are legitimate, they would not meet traditional thresholds of generalizability. Contributors were not paid and no attempt was made to recruit participants with HFpEF beyond the OPTIMISE study, this may have limited the inclusivity of the work. Lastly, we did not obtain any reflections from the PPI participants themselves on their experiences of the involvement process, so we cannot present their views nor can we ascertain whether the reflection we present resonates with them.

## Conclusions

This reflection is one of very few reports describing PPI in heart failure research. We hope that it will provide other researchers with some insight into how (or how not to) approach and deliver PPI in their research journey. To build a foundation of good practice and evidence of the potential impact of PPI in cardiovascular disease research, we need further descriptions of the involvement process and their evolution over time. PPI had a substantial effect on this project both in terms of the content of the intervention and on the researchers’ perceptions and skills of conducting meaningful PPI. Given the abundance of medical literature, it is easy to make and validate assumptions, particularly around the necessity and relevancy of a research project. As this project demonstrates, PPI that is conducted early on and in a meaningful way, can avoid assumptions about relevancy and help deliver more relevant, patient centric research.

## Data Availability

Transcribed data can be made available upon reasonable request.
